# Self-reported health and depression among EIRA cohort: a moderated mediation model of sex and perceived social support

**DOI:** 10.3389/fpsyg.2025.1540530

**Published:** 2025-06-13

**Authors:** Raquel Sánchez-Recio, Bárbara Oliván-Blázquez, Fátima Méndez-López, Santiago Gascón-Santos, Ruth Martí-Lluch, Edurne Zabaleta-Del-Olmo, Olaya Tamayo-Morales, José A. Maderuelo-Fernández, Marc Casajuana, Tomas López-Jiménez, Emma Motrico, Irene Gómez-Gómez, Álvaro Sánchez-Pérez, María Luisa Rodero-Cosano, Joan Llobera, Juan A. Bellón, Patricia Moreno-Peral, Bonaventura Bolíbar, José I. Recio-Rodríguez, Rafel Ramos, Ana Clavería

**Affiliations:** ^1^Department of Preventive Medicine and Public Health, Faculty of Social and Labor Sciences, Research Group on Health Services in Aragon (GRISSA), University of Zaragoza, Zaragoza, Spain; ^2^Zaragoza Penitentiary Center, Zaragoza, Spain; ^3^Network for Research on Chronicity, Primary Care, and Health Promotion (RICAPPS), Instituto de Salud Carlos III, Madrid, Spain; ^4^Department of Psychology and Sociology, University of Zaragoza, Zaragoza, Spain; ^5^Primary Care Research Unit (GAIAP), Health Research Institute of Aragon (IIS Aragon), Zaragoza, Spain; ^6^Department of Physiatry and Nursing, University of Zaragoza, Zaragoza, Spain; ^7^Vascular Health Research Group of Girona, Institut Universitari per a la Recerca a l'Atenció Primària Jordi Gol i Gurina (IDIAPJGol), Girona, Spain; ^8^Parc Hospitalari Martí Julià, Institut d'Investigació Biomèdica de Girona (IDIBGI), Salt, Spain; ^9^Department of Medical Sciences, School of Medicine, University of Girona, Girona, Spain; ^10^Nursing Department, Faculty of Nursing, Universitat de Girona, Girona, Spain; ^11^Fundació Institut Universitari per a la Recerca a l'Atenció Primària de Salut Jordi Gol i Gurina (IDIAPJGol), Barcelona, Spain; ^12^Gerència Territorial de Barcelona, Institut Català de la Salut, Barcelona, Spain; ^13^Universidad de Salamanca, Unidad de Investigación en Atención Primaria de Salamanca (APISAL), Instituto de Investigación Biomédica de Salamanca (IBSAL), Salamanca, Spain; ^14^Gerencia de Atención Primaria de Salamanca, Gerencia Regional de Salud de Castilla y León (SACyL), Salamanca, Spain; ^15^Departamento de Psicología Evolutiva y de la Educación, Universidad de Sevilla, Sevilla, Spain; ^16^Departamento de Métodos Cuantitativos, Universidad Loyola Andalucia, Sevilla, Spain; ^17^Unidad de Investigación Atención Primaria de Bizkaia, Subdirección para la Coordinación de la Atención Primaria, Dirección General Osakidetza, Baracaldo, Bizkaia, Spain; ^18^Grupo de Investigación en ciencias de la diseminación e implementación en servicios de salud, Instituto de Investigación Sanitaria Biocruces Bizkaia, Baracaldo, Bizkaia, Spain; ^19^Primary Care Research Unit of Mallorca, Balearic Islands Health Service (Ib-Salut), Palma, Spain; ^20^GrAPP-caIB – Health Research Institute of the Balearic Islands (IdISBa), Palma, Spain; ^21^Instituto de Investigación Biomédica de Málaga – IBIMA, Málaga, Spain; ^22^El Palo” Health Centre, Andalusian Health Service (SAS), Málaga, Spain; ^23^Department of Public Health and Psychiatry, University of Málaga (UMA), Málaga, Spain; ^24^Department of Medical Sciences, University of Girona, Girona, Spain; ^25^Primary Care Services, Catalan Institute of Health, Girona, Spain; ^26^Primary Care Research Unit, SERGAS, Galicia South Health Research Institute, Vigo, Spain

**Keywords:** social support, depression, self-reported health, health behavior and promotion, hybrid trial, primary health care

## Abstract

**Background:**

The positive relationship between health and good perceived social support has been widely demonstrated in the scientific literature. It is known that having a good social support influences the proper maintenance of health even as a protective factor, besides being a good predictor in the recovery of health during a disease process, influencing differently men and women.

**Aim:**

This project aims to study the moderating effects of perceived social support in the relationship between depression and self-perceived health according to gender, after a complex multiple-risk intervention was carried out in patients of primary health care with low social support.

**Methods:**

A cluster randomized clinical trial was developed in the subgroup of patients included in phase 3 of the EIRA project. CONSORT recommendations were followed to present the results. To determine the mediating effect between social support and self-perceived health, three regression analyses were carried out using the procedure designed by Hayes through the PROCESS macro for SPSS.

**Results:**

3,062 people (54.9% women) participated in the study. Men reported experiencing more social support and self-perceived health (*p* < 0.001) than women at the beginning of the study, but women reported higher social support at post-intervention. Moderation analyses showed that, post-intervention, those women (*b*_simple_ = −2.9867, *p* < 0.001) and males (*b*_simple_ = −1.4337, *p* < 0.001) who scored lower in depression reported higher social support.

**Conclusion:**

In primary care, it is necessary to encourage intervention strategies that promote social networks as a key element of positive action aimed at maintaining and improving the population’s health, especially in adults and more specifically in women.

**Clinical trial registration:**

ClinicalTrials.gov, identifier NCT03136211.

## 1 Introduction

Interpersonal relationships among individuals within various settings (family, work, etc.) emerge as crucial elements for their health, as they will ultimately determine the existence of problems or, conversely, wellbeing (Fachado et al., 2013). Perceived social support is a relatively recent concept when associated with stress and health. Currently, there is no consensus on its definition, although that of Thoits (1982, 1992) is the most widely spread and clear. They define social support as “the extent to which a person’s basic social needs are met through interaction with others.” These basic needs include affection, belonging, identity, security, and approval. They are typically fulfilled by support or social network, which typically involves family and friends although it is not limited to them. Nonetheless, the existence of a support network does not guarantee the presence of adequate social support within it (Barrón, 1990), since it is a complex, ambiguous, and multidimensional concept which is subject to various interpretations that encompass aspects related to social networks, family bonds, social integration, marital status, social class, or attendance at religious services, among others (Fachado et al., 2013).

Besides this, there are gender differences in the way social support is perceived. Women have generally more social support, that is, they have more people close to them than men, but men have broader social networks, even if their relationships are not so deep (Fuhrer et al., 1999). Women experience differences in socialization as well as different social roles that lead them to create more emotionally supportive ties, mobilize more social support in times of stress, and provide more frequent and timely social support to others than men (Hosseini et al., 2021).

According to the literature, the effects of perceived social support on health can occur in two ways: either by mitigating the harmful effects of social stressors and the disease itself or, in the opposite case, its absence causes a stress factor that affects one’s health (Cubeiro, 2021). The meta-analysis by Holt-Lunstad et al. (2010) already demonstrated the influence of social relationships on the risk of death. The influence of social relationships is comparable to that other well-established risk factors for mortality, such as smoking and alcohol consumption, and surpasses the influence of other factors, such as physical inactivity and obesity. In this line, others authors (Holt-Lunstad et al., 2010; Wang et al., 2018), showed, that low perceived social support was associated with higher mortality rates and was comparable to other risk factors such as obesity, physical inactivity, and smoking. Various studies confirm the influence of perceived social support on people’s quality of life, with higher levels in those individuals with a strong social support, and emphasizing the importance of family support (Alfonso Figueroa et al., 2016; Gariépy et al., 2016). On the other hand, social support is also related to depressive symptoms and/or depression. Several mechanisms have been proposed by which better social support could contribute to reducing depression; for example, perceiving oneself as part of a support network has been linked to various positive health outcomes (Buckman et al., 2021). As seen in the systematic review by Gariépy et al. (2016), spousal support was the source of social support most consistently associated with protecting against depression in adults (100% of studies reported a significant association), followed by family support (88% of studies), friends (73% of studies), and children (67% of studies). Social networks are not only an important source for fulfilling social and self-esteem needs (10), but also for community integration and participation, which have a significant impact on quality of life and wellbeing (Arias, 2013). However, both the relationship between social support and depression, as well as the relationship between depression and health perception, and social support and health perception, are complex and may have bidirectional effects (Kim et al., 2025). Moreover, in studying these effects, gender differences must also be considered, both in the perception of social support (Hosseini et al., 2021) and in the prevalence of depression (Stanton et al., 2025). This makes it necessary to delve deeper into the reciprocal relationships between these concepts.

There is a growing interest in the effects of social relationships on health and their policy and delivery implications, since perceived social support has been observed to be modifiable. Therefore, it is necessary to consider this for the development of preventive interventions and make it a priority area for healthcare professionals and health policymakers (Buckman et al., 2021). Complex and multi-risk interventions could enhance the strengthening and creation of support networks through support groups. These strategies may include individual, group, and community approaches that could be combined. Nevertheless, more long-term clinical trials are needed to address different lifestyles and generate more scientific evidence on these types of interventions (Alvarez-Bueno et al., 2015; Hoogeveen et al., 2015; Mc Sharry et al., 2015). Leveraging new approaches to health interventions such as social networks could be beneficial given their outreach capacity among the population (Petkovic et al., 2021).

In this context, the Research Network on Preventive Activities and Health Promotion (redIAPP) (Red de Investigación en Actividades Preventivas y Promoción de la Salud, n.d.) began designing a complex multi-risk intervention for the population aged between 45 and 75 in 2012 to promote health behaviors, improve their quality of life, prevent the most common chronic diseases and their complications, and contribute to an active and healthy aging (Edurne et al., 2018; Aznar-Lou et al., 2021; Gómez-Gómez et al., 2023). Within this project, we aimed to explore how a mediating variable—in this case, perceived social support—influences changes in other variables using a causal analysis model (Hernan and Robins, 2020).

Thus, the objective of this research is to evaluate the sex moderating effect of perceived social support on the relationship between depression and self-perceived health, following a complex multiple-risk intervention carried out with patients in primary care (the EIRA cohort).

The conceptual hypothesis of the presented model would be that better self-perceived health would lead to less self-perceived depressive symptoms, but this relationship would be mediated by perceived social support, which in turn would be moderated by gender.

## 2 Materials and methods

### 2.1 Design

Cluster randomized controlled trial was used, which is included in Phase 3 of the EIRA project (Edurne et al., 2018). The trial registration number is NCT03136211. The CONSORT guidelines were followed to present the results (Boutron et al., 2017).

### 2.2 Scope

This study is part of the EIRA study, which was conducted in 26 Primary Health Care Centers (PHCC) located in seven Health Departments in Spain, from January 2016 to December, 2019. The different PHCCs are composed of a multidisciplinary team of doctors, nurses, pediatricians, midwives, social workers, pharmacists, and dentists who provide healthcare, health education, health promotion, and disease prevention activities for the community.

### 2.3 Inclusion and exclusion criteria

The selected PHCCs had to meet the following criteria: not being in areas with much sociocultural diversity or in areas with considerable tourism, having access to the internet, offering the possibility of prescribing community activities, recommending community activities and having available professionals particularly committed to this study (professionals’ participation was voluntary).

### 2.4 Patients

All patients within the EIRA study, aged 45–75, who exhibited two or more unhealthy behaviors (tobacco consumption, non-adherence to the Mediterranean diet, or low physical activity levels) were included. Exclusion criteria are specified in the study protocol (Edurne et al., 2018).

### 2.5 Intervention (EIRA study)

An educational intervention to promote health about Mediterranean food, physical activity and smoking cessation that lasted 12 months took place in the PHCCs and was personally administered by patients’ doctors and nurses.

The professionals who participated in this intervention were trained for more than 60 h to standardize the intervention.

The EIRA intervention consisted in a multicomponent approach for each life habit at three levels (individual, group and community) tailored to each patient’s stage of conduct change (Bully et al., 2015). The individual intervention consisted in a short educational assistance during consultations and sending reminder text messages. Those people in the contemplative and preparation phase could also employ a mobile application to perform continuous activity toward physical exercise and diet. Group interventions consisted in an organized workshop which focused only on physical activity and diet. Finally, the community intervention included recommending community resources to help improve adherence to the Mediterranean diet and increase physical activity (social prescription) (Bully et al., 2015).

While the intervention was underway, health care professionals attempted to adapt the different intervention components to each patient’s characteristics (resources, expectations, requirements, etc.). During the first visit, professionals agreed with patients on a specific approach and follow-up plan.

The individual intervention included two to three visits and the possibility of further reinforcing. Depending on the change phase in which each patient was found regarding their lifestyle habits, the following took place: (1) “a very brief intervention” with the objective to raise more awareness of the need to change life habits and support any changes or help to prevent possible relapses; (2) “a brief intervention” to establish a specific agreed plan to change behavior. Health care professionals performed this brief intervention by applying motivational techniques after receiving 20 h of online training (Fontán et al., 2013). This intervention type was supported by an informative website, the sending of personalized texts and the use of mobile applications or other electronic devices (pedometers, smart watches, etc.). At the end of consultations, the professionals involved handed out to each patient the written support material as a reinforcement mechanism.

The group intervention took place during a health education workshop and focused on healthy diet and physical activity. It aimed to reinforce the recommendations made in the individual intervention, and to provide patterns that helped follow the Mediterranean diet and practice physical exercise. This workshop began after the first individual intervention visit. It included two sessions lasting 90–120 min each, performed by the health care professionals from the PHCCs.

The community intervention consisted of recommending social activities (Herdman et al., 2001) in the patient’s own neighborhood according to the detected risky life habits and accessibility to community resources; e.g., gyms, gardens, healthy walking, etc. The professionals from the PHCCs previously identified community’s health assets (Manea et al., 2015) and selected the most appropriate ones according to the detected unhealthy behaviors, accessibility and the possibility of referring participants.

All the details of interventions are specified in the study protocol (Edurne et al., 2018). No blinding was included in relation to either the patients in the two study groups (control group and intervention group) or the health care professionals who undertook the EIRA intervention.

### 2.6 Recruitment

Population selection was performed opportunistically between February 2017 and January 2018 and was stratified by the age/sex distribution expected from the participating PHCCs.

The recruitment was performed by doctors and nurses from the PHCCs through the different methods set out in the study protocol: (1) During the visits that are part of usual health care; (2) Self-administered questionnaires handed out in waiting rooms or at admission desks; (3) Advertising on posters inside PHCCs and; (4) Telephoning the selected patients by revising electronic medical records. The professionals were previously trained to ensure a homogenous procedure.

### 2.7 Instruments

#### 2.7.1 Perceived social support

Perceived Social Support was assessed with the Functional Social Support Questionnaire (DUKE-UNC-11) (Broadhead et al., 1989; Bellón et al., 2000; Herdman et al., 2001). This tool is used to examine social support in two dimensions: affective support and confidential support. It consists of a total of 11 items with 5 response alternatives ranging from *much less than I want* to *as much as I want.* A score equal to or higher than 32 indicates normal support, while a score lower than 32 indicates low perceived social support. It has shown good psychometric properties for the Spanish population (Bellón et al., 2000), presenting a Cronbach’s alpha coefficient for the total scale of 0.90, and 0.88 and 0.79 for the subscales of confidential support and affective support, respectively. The DUKE-UNC-11 was administered both at baseline and at 10-14-month follow-up.

#### 2.7.2 Self-perceived health

It was measured by EuroQol-5D (EQ-5D) questionnaire (Herdman et al., 2001). The EQ-5D is a generic instrument to measure quality of life. It is a self-administered instrument in which the person assesses his or her state of health. It is especially useful in primary care. In the first part, this instrument contains five health dimensions (mobility, self-care, activities of daily living, pain/discomfort and anxiety/depression), each of them having three levels of severity (no problems, some problems or moderate problems, and more severe problems). Its second part consists of a 20 cm vertical, Visual Analogue Scale (VAS) ranging from 0 (*worst unimaginable state of health*) to 100 (*best imaginable state of health*). On this scale, the individual must select the score that best reflects the assessment of his or her global state of health at the time of completing the questionnaire. This instrument has good psychometric properties in the Spanish population and even in different circumstances and pathologies.

#### 2.7.3 Self-perceived depression

The Patient Health Questionnaire-9 (PHQ-9) was used to determine the prevalence of depression symptoms (Manea et al., 2015). The PHQ-9 is a self-report measure of depression consisting of nine items matching the Diagnostic and Statistical Manual of Mental Disorders Fourth Edition (DSM-IV) criteria of major depression. Respondents are asked to rate each of the items on a scale of 0 to 3 on the basis of how much a symptom has bothered them over the last 2 weeks (0 = *not at all*, 1 = *several days*, 2 = *more than half the days*, 3 = *nearly every day*). Data from the PHQ-9 primary care study showed that the algorithm had a sensitivity of 73% and a specificity of 98%. In the validation study of the summed-item method, a score ≥10 had a sensitivity of 88% and a specificity of 88% for major depressive disorder.

#### 2.7.4 Sociodemographic variables

Age, marital status, occupational level and education were obtained from electronic medical record of Primary Health Care (PHC) of the research team in each autonomous community.

#### 2.7.5 Comorbidities

Comorbidities were measured through the Charlson Comorbidity Index (CCI) (Charlson et al., 2022). The CCI is the most widely used measure of comorbidity due to its ease of use (it is scored a continuous variable). It consists of 19 elements corresponding to medical conditions, which are weighted to provide a total score for the sum of the different pathologies.

### 2.8 Variables

The outcome variable was self-related depression and the criterion variable was self-perceived health on the EIRA Study. Perceived social support was included as the mediating variable, and sex as the moderating variable.

Regarding covariables, we considered age grouped in three categories (45–54 years, 55–64 years, ≥65 years), marital status grouped in two categories (married/living with a partner and not married/not living with a partner), occupational level grouped in five categories (working, unemployed, retired, unpaid household labor and others), education grouped in four categories (high, medium, low education and without studies) and CCI.

### 2.9 Data analysis

Following the CONSORT recommendations for non-pharmacological interventions and, in order to reduce any bias due to lack of data during the follow-up, an intention-to-treat analysis was carried out.

After sorting and analyzing the quality of the obtained data, multiple imputation was performed by random forest to avoid the collinearity influence. Missing values were assumed to be missing at random. The following were included: intervention (yes/no), PHCCs, all the initial/final outcome variables, stratification variables (age, sex), auxiliary variables with losses below 30% and the possible influence on outcome (comorbidities, motivation, clinical variables). The imputation phase created many copies of datasets, and each one contained different estimations of missing values. Then, the imputed datasets were analyzed.

A descriptive analysis (frequencies for categorical variables; means and standard deviation for continuous variables) was performed to examine the composition of the sample, and the normality or non-normality of the data was tested using the Kolmogorov–Smirnov test with the Lilliefors modification, since the sample exceeded 30 cases. Secondly, in order to see if there were differences between men and women in the study variables, mean differences were studied using Student’s t-test for quantitative variables and chi-squared test for qualitative variables.

### 2.10 Mediation/moderation analysis

**Figure 1:**
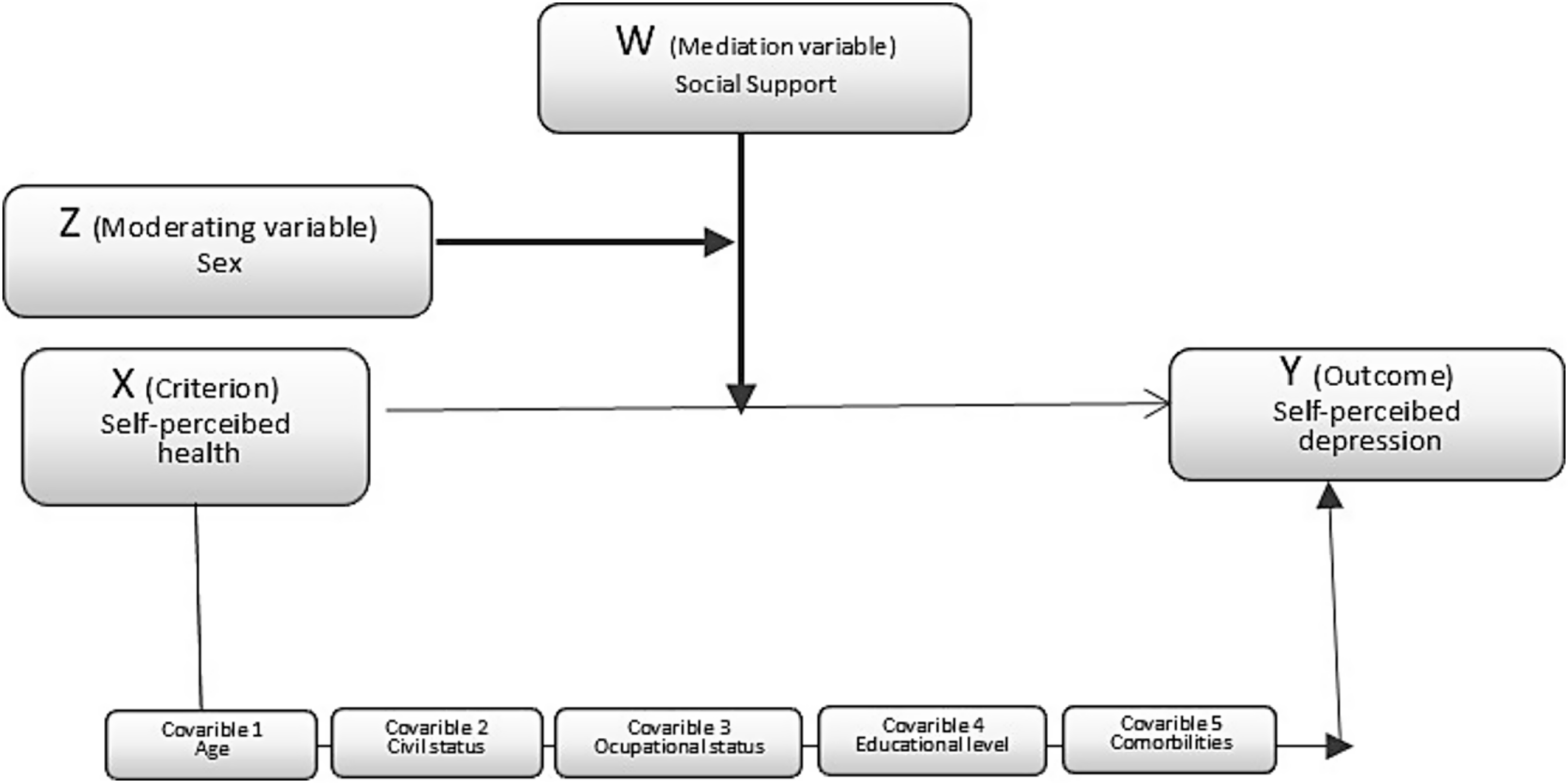
Conceptual mediation/moderation model (model 4 Process).

A mediation analysis through an educational intervention (EIRA Study) was conducted using the Process macro for SPSS, with the aim of exploring the perceived social support as an intermediate mechanism that explains changes in health outcomes (self-perceived depression and self-perceived health) and of confirming how sex acts as confounder or mediator in this association (Figure 1). To determine the mediating effect, various regression analyses were developed using the procedure designed by Baron and Kenny (1986). This procedure requires the predictor, criterion and mediating variables (perceived social support) to be positively correlated with each other. Given the positive associations observed between health and the covariables (age, marital status, occupational level, education and CCI), we decided to control the effects of these variables by introducing them in the first step of the regression. The bootstrapping technique with 10,000 subsamples was used to estimate the confidence interval (95%).

Tests were considered significant when *p* was <0.05. The analyses were carried out using IBM SPSS Statistics 24^®^ and Stata 14^®^, both licenses from the University of Zaragoza, Spain. Since the microdata from the surveys are public and anonymous, ethical approval was not required for this study.

## 3 Results

### 3.1 Descriptive statistics

Statistics for all variables are presented in Table 1. 3,062 people (54.9% women) participated in the study. For both sex, the most representative age group was 45–65 years old (37.1% men vs. 40.1% women). 72% of men reported being married compared to 65.3% of women (*p* < 0.001). 47.2% of men and 43.7% of women referred to be working, while 21.7% women stated to be unpaid household workers vs. 0.7% in men. Close to 40% of men and women had medium and low education. Finally, around 30.1% of men and 22.1% of women (*p* < 0.001) reported suffering from one comorbidity.

**Table 1:** Baseline characteristic of the patients included in the study.

Variables			Men (1,381, 45.1%)	Women (1,681, 54.9%)	Total (3,062, 100%)	*p* (Chi^2^)
Sociodemographic	Age	45–65 years	512 (37.1%)	674 (40.1%)	1,186 (38.7%)	0.112
		55–64 years	486 (35.2%)	591 (35.2%)	1,077 (35.1%)	
		≥65 years	383 (27.7%)	416 (24.7%)	799 (26,0%)	
	Marital status					
		Married / Living with a partner	995 (72.0%)	1,098 (65.3%)	2,083 (68.4%)	<0.001
		No marries/ Not living with a partner	386 (28.0%)	583 (34.7%)	969 (31.6%)	
						
	Occupational Status					
		Working	651 (47.2%)	734 (43.7%)	1,385 (45.2%)	<0.001
		Unemployed	150 (10.9%)	138 (8.2%)	288 (9.4%)	
		Retired	480 (34.6%)	330 (19.6%)	810 (26.5%)	
		Unpaid household labor	9 (0.7%)	365 (21.7%)	374 (12.2%)	
		Others (studying, pensioners, etc.)	91 (6.6%)	114 (6.8%)	205 (6.7%)	
	Education					
		High	236 (17.2%)	278 (16.7%)	514 (16.9%)	0.873
		Medium	547 (39.9%)	647 (38.9%)	1,194 (39.4%)	
		Low	510 (37.2%)	636 (38.3%)	1,146 (37.8%)	
		Without studies	78 (5.7%)	101 (6.1%)	179 (5.9%)	
	Charlson Index	1	415 (30.1%)	372 (22.1%)	787 (25.7%)	<0.001
		3	24 (1.7%)	6 (0.4%)	30 (0.9%)	
		5	1 (0.1%)	0 (0%)	1 (0.03%)	
		7	1 (0.1%)	0 (0%)	1 (0.03%)	

In relation to the main variables of this study, Table 2 shows these pre-and post-intervention descriptive analyses. In terms of self-perceived health, men reported better self-perceived health than women before and after the intervention (*p* < 0.001). When analyzing self-perceived depression, women indicated higher levels compared to men, but this difference was only significant at post-intervention (*p* < 0.001). Finally, regarding perceived social support, men reported more perceived social support than women in the pre-intervention assessment, but women showed higher scores at post-intervention, with differences statistically significant in both cases. During the intervention, some improvement was observed in two health outcomes for both sex (self-perceived health, self-perceived depression *p* < 0.001). However, in relation to perceived social support, women did show a statistically significant improvement (*p* = 0.019), which was not observed in men (*p* = 0.081).

**Table 2:** Descriptive pre and post-intervention analysis of the outcome variables.

Variables	Pre-intervention				Post-intervention			
	Total (M, SD)	Men (M, SD)	Women (M, SD)	*p* (Chi2)	Total (M, SD)	Men (M, SD)	Women (M, SD)	*p* (Chi^2^)
Self-perceived health	0.81 (0.19)	0.84 (0.18)	0.791 (0.20)	<0.001	0.87 (0.17)	0.89 (0.15)	0.86 (0.19)	<0.001
Self-perceived depression	4.96 (5.72)	3.93 (5.03)	5.82 (6.11)	0.08	4.95 (5.77)	4.38 (5.57)	5.42 (5.88)	<0.001
Social support	45.09 (8.97)	45.77 (8.57)	44.53 (9.19)	0.01	46.42 (9.81)	46.29 (10.20)	46.53 (9.46)	0.02

M, Mean; SD, Standard Deviation.

### 3.2 Testing for mediating effects

As already established, to test that perceived social support would mediate the link between self-perceived health and self-perceived depression, PROCESS macro (Model 4) in SPSS was conducted pre-and post-intervention (Table 3). Age, marital status, occupational status, education and CCI were entered as covariates in the analysis. As Table 3 for the pre-intervention and Table 4 for the post-intervention illustrate, self-perceived health was positively linked to self-perceived depression: *b_pre-intervention_* = 10.6401, *b_post-intervention_* = 8.9243, *p* < 0.001 (Model 1). However, self-perceived health was negatively linked to social support: *b_pre-intervention_* = −12.0410, *b_post-intervention_* = −2.6278, *p* < 0.001 (Model 2), and social support was also negatively related to self-perceived depression: *b_pre-intervention_* = −13.8890, *b_post-intervention_* = −4.0928, *p* < 0.001 (Model 3).

**Table 3:** Mediation analysis (Model 4 Process).

Predictors	Model 1		Model 2		Model 3	
	*b*	*t*	*b*	*t*	*b*	*t*
Self-perceived health	10.64	12.93***	−12.04	−25.81***	−13.89	−29.18***
Perceived social support			−0.17	−17.37***		
Age	0.29	1.22	−0.59	−4.45***	−0.64	−4.59***
Marital status	−2.44	−7.24***	0.19	1.03	0.62	16,043.9**
Occupational status	0.13	0.95	0.19	2.63**	0.17	2.24**
Education	0.17	0.33	−0.25	−1.38	−0.28	−1.47
Comorbidities	−0.25	0.23	0.29	0.77	0.08	0.61
*F*	41.55		195.02		161.32	
*R* ^2^	0.07		0.31		0.24	

Outcome variable self-perceived depression, mediation perceived social support (EIRA Study-pre-intervention).

*p < 0.10, **p < 0.005, ***p < 0.0001. Model 4 PROCESS SPSS. Model 1 outcome variable: Social Support. Model 2 outcome variable: SPH.

**Table 4:** Mediation analysis (Model 4 Process).

Predictors	Model 1		Model 2		Model 3	
	*b*	*t*	*b*	*t*	*b*	*t*
Self-perceived health	8.92	8.86***	−2.63	−4.48***	−4.09	−6.94***
Perceived social support			−0.16	−16.09***		
Age	1.01	3.70**	0.86	−5.57***	−1.02	6.39***
Marital status	−0.37	−1.45	0.40	2.78**	0.46	3.08**
Occupational status	−0.24	−1.58	0.13	1.48	0.17	1.87**
Education	0.33	1.48	0.61	4.88***	0.56	4.27***
Comorbidities	0.33	1.18	−0.24	−1.52	−0.29	−1.79*
*F*	17.02		55.56		19.96	
*R* ^2^	0.03		0.11		0.04	

Outcome variable self-perceived depression, mediation perceived social support (EIRA Study-post-intervention).

*p < 0.10, **p < 0.005, ***p < 0.0001. Model 4 PROCESS SPSS. Model 1 outcome variable: Perceived Social Support. Model 2 outcome variable: SPH.

The indirect effect of perceived social support on the extent of self-perceived depression via self-perceived health was *b_pre-intervention_* = −1.8480 (SE = 0.2162, 95%CI = [−2.2938, −1.4359]), and *b_post-intervention_* = −1.4651 (SE = 1.973, 95%CI = [−1.4651, −1.0878]). 95%CI does not include zero, indicating that social support did mediate the relationship between self-perceived health and self-perceived depression.

### 3.3 Moderating role that sex post-intervention

As for assessing the potential moderating role that sex might have in the mediation model, PROCESS macro (Model 59) by Hayes was used (Hayes and Preacher, 2014). According to Table 5, the interplay between self-perceived health and sex at the pre-intervention period was negative and significant. At post-intervention this same interaction changed, showing a positive, significant relationship (*b*_simple_ = 0.3629, *p* < 0.05). Furthermore, the association between sex and perceived social support was nonsignificant at pre-and post-intervention.

**Table 5:** Testing the moderating role of sex in the mediation model (model 59 Process).

Predictors	Pre-intervention				Post-intervention			
	Model 1		Model 2		Model 1		Model 2	
	*b*	*t*	*b*	*t*	*b*	*t*	*b*	*t*
Self-perceived health (SPH)	10.37	3.73**	−7.76	1.52***	11.38	3.38**	−2.02	−6.89***
Perceived social support (PSS)			−0.15	−4.51***			−0.19	−3.41**
Sex	−0.83	−0.59	3.61	3.48**	0.71	0.42	−0.34	−1.69*
Sex*SPH	0.26	0.16	−2.28	−2.51**	−1.19	−0.61	0.36	2.13**
SS*Sex			−0.02	0.89			0.01	0.56
Age	0.35	1,44	−0.64	−4.87***	0.66	2.47**	−0.07	−2.85**
Marital status	0.24	−1.95	0.17	2.59**	−0.44	−1.74*	0.01	0.60
Occupational status	0.12	0.91	0.18	2.46**	0.01	0.09	0.03	2.10**
Education	0.04	0.19	−0.09	−0.88	0.12	0.55	0.03	1.84*
Charlson index	−0.13	−0.52	0.07	0.49	−0.37	−1.30	0.01	0.12
*F*	74.57		144.74		67.77		55.26	
*R* ^2^	0.05		0.32		0.05		0.19	

Sex is codified 0 female and 1 male. *p < 0.10, **p < 0.05, ***p < 0.001. Model 59 PROCESS SPSS. Model 1 outcome variable: Perceived Social Support. Model 2 outcome variable: SPH.

**Figure 2:**
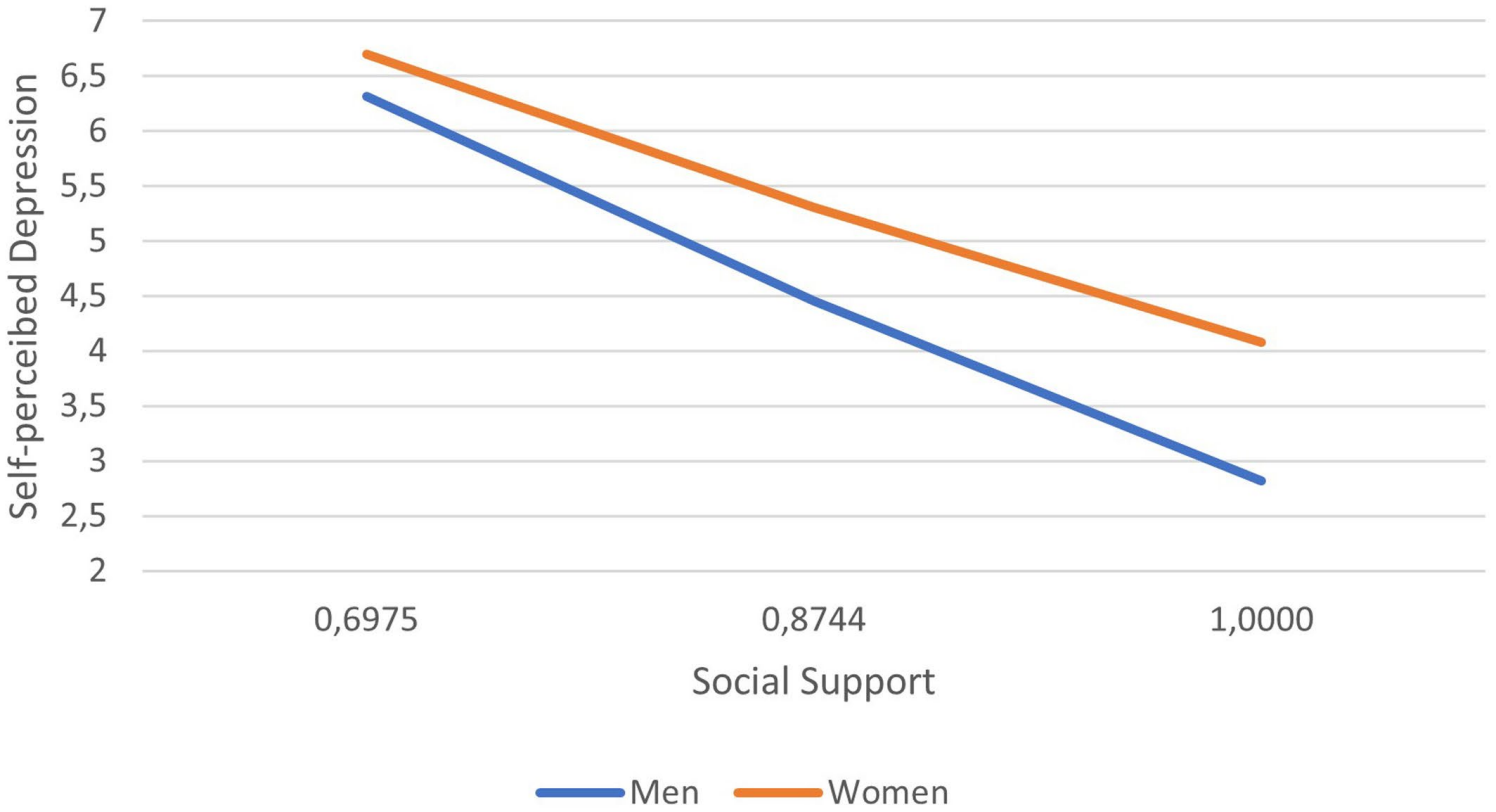
Social support according to self-perceived depression, shown separately for men and women at pre-intervention time.

**Figure 3:**
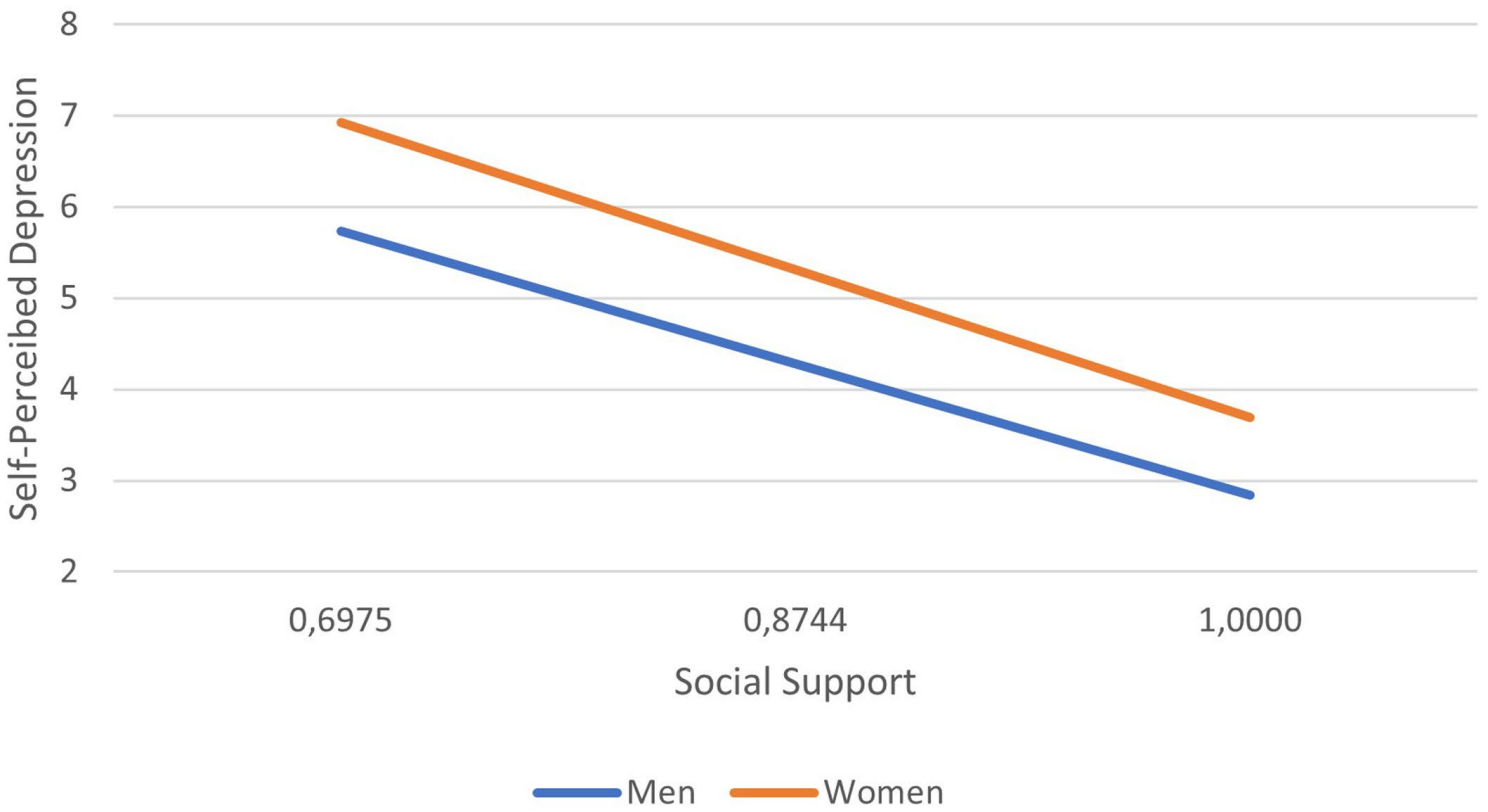
Social support according to self-perceived depression, shown separately for men and women at post-intervention time.

As observed, sex moderated the relationship between perceived social support and self-perceived depression both at pre- and post-intervention, although it changed from being negative before the intervention to being positive after it. Considering the descriptive purpose, we plotted the predicted social support according to self-perceived depression separately for men and women (Figure 2 pre-intervention, Figure 3 post-intervention). It was observed that, for both cases and both pre-and post-intervention, higher scores in depression related to lower scores in perceived social support and that, conversely, the higher the perception of social support, the lower the score in depression. Post-intervention, differences were also noted between men and women, the latter continuing to show more social support. The gap between both sex is greater when both men and women report having good social support, since women are the ones who have the best results in perceived depression. Finally, a simple regression test indicated that, post-intervention, women’s lower levels of self-perceived depression were related to lower levels of social support *b*_simple_ = −2.9867, *p* < 0.001. For males (post-intervention), the association between self-perceived depression and perceived social support was similar to females, but much weaker *b*_simple_ = −1.4337, *p* < 0.001.

## 4 Discussion

Depression is considered to be the principal cause of disability worldwide and one of the major social health costs (Sobocki et al., 2006), and by 2030, it is expected to be the leading cause of morbidity in the world (Gabilondo Cuéllar et al., 2010; Ferrari et al., 2013; Depression, 2017). These data must encourage us to direct our efforts toward the prevention and management of this mental pathology, and the results of this study may contribute to this goal.

Our research presents different health outcomes, both in self-perceived health and depression, in men and women, with women exhibiting poorer health results at the two time points analyzed (pre- and post-intervention). Following the intervention, particularly in the case of women, we observed an improvement in both self-perceived health and depression.

The gender gap in self-perceived health and depression has a multifactorial etiology (Hyde et al., 2008; Salk et al., 2017), but it is relevant when taking into account that gender differences in depression occur in a developmental context (Salk et al., 2017). Certain theories highlight how specific vulnerability factors are activated at critical periods of development and/or interact with stressors at certain times in the life cycle to produce this gender difference. Common major life transitions often cause stress and anxiety (and affect women more than men), and these transitions lead to role changes that may be considered positive, negative or neutral. In line with this, gender differences are observed, including giving birth/having a child, dependent care, double working shifts, menopause, retirement, among others, which highlights the existence of differential gender roles for men and women today (Arcand et al., 2020; Jaehn et al., 2020; Moustafa et al., 2020; Lin et al., 2021).

Similarly, both men and women had higher scores in perceived social support at both pre and post-intervention, with scores improving in both genders after the intervention. Throughout history, a number of studies have shown that there are differences in the perception of social support between men and women (Fusilier et al., 1986; Ganster and Victor, 1988; Häusser et al., 2023). Furthermore, it has been demonstrated that gender roles play a significant role in men’s perception of social support, which comes primarily from their workplace and social interactions outside the household, while women’s perception of social support mainly stems from support within the household and from family. This can help explain the differences found in this study and how the intervention has improved women’s perception of social support by increasing their social network outside of the family and their home environment.

The association between perceived social support and self-perceived health has already been reported in different studies. Individuals with better self-perceived health report greater social support (Lino et al., 2013; Kim et al., 2016). Similarly, individuals who are unhappy with their social network are two to four times more likely to rate their health as poor, especially in the most underprivileged groups (Lima-Costa et al., 2005; Surkan et al., 2009; Kumparatana et al., 2017).

There is an abundant bibliography establishing the relationship between depression and social support, and specifically demonstrating the importance of lack of relationships and social support during the onset and development of depression (Lorant et al., 2003; Plaisier et al., 2007; Golden et al., 2009; Lino et al., 2013). But on the other hand there is also a bibliography that shows a relationship between depression and low self-perceived health (Castro-Costa et al., 2008).

Likewise, the relationship between depression, perceived social support and self-perceived health is complex, and it reflects the individual’s integrated perception of the biological, psychological and social dimensions of health (Jylhä, 2009). The relationship between social support and depression can be considered bidirectional, but so can the relationship between health perception and depression. That is, the lack of social support increases the likelihood of developing depression, but depression can also lead to a reduction in social skills and isolation (Thompson et al., 2015). Moreover, it is important to consider in this bidirectionality the difference between actual social support and perceived social support, and their relationship with mental health. Perceived social support is inversely related to the symptoms of depression and mental illness, meaning that the more severe the mental illness, the less perceived social support there is (Leskelä et al., 2004). The relationship between self-perceived health and depression can also be considered bidirectional. Several studies show that both depression and depressive symptoms are a risk factor for both self-perceived health and for diseases such as diabetes, among others (Nicolosi et al., 2011). Medical illness is a risk factor for depression, and it has even been found to have a stronger correlation with depression than with other variables (Payne et al., 1993; Geerlings et al., 2000; Sa, 2003; de Jonge et al., 2006; Koster et al., 2006). In fact, there are diseases that double the risk of developing depression, while others can quadruple it (Odds Ratio = 1.2–4.5). The strongest association occurs when depression is coupled with anxiety (Scott et al., 2007).

The results of this study shed light into the role of social support acting as a moderator in the relationship between depression and self-perceived health. This study also demonstrated gender differences, with women having a worse self-perception of health, and more depression than men. Social support can be an asset for health to improve the gender disparity because women with better scores in social support, also have lower depression levels.

Other studies have also tried to clarify the intimate relationship between self-perceived health, social support and gender. In line as our findings, Sun et al. (2024) observed the relationship between these factors, finding that high self-perceived health correlates with non clinically significant scores of depression in men and with non clinically significant scores of depression and better social integration in women.

On the contrary, Lino et al. (2013) established the association between self-perceived health and social support shown by bivariate analyses, which was attenuated when a multivariate analysis was carried out. This may be explained by depression acting as a confounding factor, but these results should be treated cautiously since this study was based on 180 elderly subjects.

This study has certain limitations, mainly arising from the complexity of establishing a theoretical framework and imbricated relationship between perceived social support, depression and self-perceived health. Another limitation is that self-reported data were used for measuring the studied data, although there is still a good correlation with clinical health data. The final limitation of this study is that the social support collected is the *perceived* one. On another note, the main strengths of this study are the population and the great sample size. The sample population are patients of PHC aged between 45 and 75. This is relevant since most similar studies have been carried out in older adults (60 years and older). Our sample size reached 3,062 participants, 1,381 of them being men and 1,681 women, which allowed us to obtain robust results, also comparing by gender. These strengths increase the heterogeneity of the sample and bring it closer to the real population attended by PHC professionals.

## 5 Conclusion

In conclusion, interventions developed in PHC improve perceived social support, decrease depression and increase self-perceived health, its effect being higher in women. Therefore, on the basis of these results, policy makers, clinicians and researchers could take action by developing intervention programs that facilitate friendships and social support, mainly in women, in order to improve mental health and self-perceived health.

## Data Availability

The raw data supporting the conclusions of this article will be made available by the authors without undue reservation.

## References

[ref1] Alfonso Figueroa L. Soto Carballo D. Santos Fernández N. A. (2016). Calidad de vida y apoyo social percibido en adultos mayores. Revista de Ciencias Médicas de Pinar del Río 20. Available at: http://scielo.sld.cu/scielo.php?script=sci_arttext&pid=S1561-31942016000100012&lng=es&tlng=es

[ref2] Alvarez-Bueno C. Cavero-Redondo I. Martinez-Andres M. Arias-Palencia N. Ramos-Blanes R. Salcedo-Aguilar F. (2015). Effectiveness of multifactorial interventions in primary health care settings for primary prevention of cardiovascular disease: a systematic review of systematic reviews. Prev. Med. (Baltim) 76, S68–S75. doi: 10.1016/j.ypmed.2014.11.028, PMID: 25511466

[ref3] Arcand M. Juster R.-P. Lupien S. J. Marin M.-F. (2020). Gender roles in relation to symptoms of anxiety and depression among students and workers. Anxiety Stress Coping 33, 661–674. doi: 10.1080/10615806.2020.1774560, PMID: 32490683

[ref4] Arias C. J. (2013). El apoyo social en la vejez: la familia, los amigos y la comunidad. Revista Kairós-Gerontologia 16, 313–329. doi: 10.23925/2176-901X.2013v16i4p313-329

[ref5] Aznar-Lou I. Zabaleta-del-Olmo E. Casajuana-Closas M. Sánchez-Viñas A. Parody-Rúa E. Bolíbar B.. (2021). Cost-effectiveness analysis of a multiple health behaviour change intervention in people aged between 45 and 75 years: a cluster randomized controlled trial in primary care (EIRA study). Int. J. Behav. Nutr. Phys. Act. 21, 1–15. doi: 10.1186/s12966-024-01674-8, PMID: 39654047 PMC11626747

[ref6] Baron R. M. Kenny D. A. (1986). The moderator–mediator variable distinction in social psychological research: conceptual, strategic, and statistical considerations. J. Pers. Soc. Psychol. 51, 1173–1182. doi: 10.1037/0022-3514.51.6.1173, PMID: 3806354

[ref7] Barrón A. (1990). Apoyo social: definición. Jano 38, 62–73.

[ref8] Bellón J. Á. Lardelli P. Luna J. D. Delgado A. (2000). Validity of self reported utilisation of primary health care services in an urban population in Spain. J. Epidemiol. Community Health 54, 544–551. doi: 10.1136/jech.54.7.544, PMID: 10846198 PMC1731703

[ref9] Boutron I. Altman D. G. Moher D. Schulz K. F. Ravaud P. for the CONSORT NPT Group* (2017). CONSORT statement for randomized trials of nonpharmacologic treatments: a 2017 update and a CONSORT extension for nonpharmacologic trial abstracts. Ann. Intern. Med. 167, 40–47. doi: 10.7326/m17-0046, PMID: 28630973

[ref10] Broadhead W. E. Gehlbach S. H. deGruy F. V. Kaplan B. H. (1989). Functional versus structural social support and health care utilization in a family medicine outpatient practice. Med. Care 27, 221–233. doi: 10.1097/00005650-198903000-00001, PMID: 2784523

[ref11] Buckman J. E. J. Saunders R. O’Driscoll C. Cohen Z. D. Stott J. Ambler G.. (2021). Is social support pre-treatment associated with prognosis for adults with depression in primary care? Acta Psychiatr. Scand. 143, 392–405. doi: 10.1111/acps.13285, PMID: 33548056 PMC7610633

[ref12] Bully P. Sánchez Á. Zabaleta-del-Olmo E. Pombo H. Grandes G. (2015). Evidence from interventions based on theoretical models for lifestyle modification (physical activity, diet, alcohol and tobacco use) in primary care settings: a systematic review. Prev. Med. (Baltim) 76, S76–S93. doi: 10.1016/j.ypmed.2014.12.020, PMID: 25572619

[ref13] Castro-Costa É. Lima-Costa M. F. Carvalhais S. Firmo J. O. A. Uchoa E. (2008). Factors associated with depressive symptoms measured by the 12-item general health questionnaire in community-dwelling older adults (the Bambuí health aging study). Brazil. J. Psychiatry 30, 104–109. doi: 10.1590/s1516-44462008005000007, PMID: 18470408

[ref14] Charlson M. E. Carrozzino D. Guidi J. Patierno C. (2022). Charlson comorbidity index: a critical review of clinimetric properties. Psychother. Psychosom. 91, 8–35. doi: 10.1159/000521288, PMID: 34991091

[ref15] Cubeiro M. T. (2021). Ignorancia y complejidad: La sociología de la enfermedad mental. Acciones e Investigaciones Sociales 42, 43–75. doi: 10.26754/ojs_ais/ais.2021426228

[ref16] de Jonge P. Kempen G. I. J. M. Sanderman R. Ranchor A. V. van Jaarsveld C. H. M. van Sonderen E.. (2006). Depressive symptoms in elderly patients after a somatic illness event: prevalence, persistence, and risk factors. Psychosomatics 47, 33–42. doi: 10.1176/appi.psy.47.1.33, PMID: 16384805

[ref17] Depression W. H. O. (2017). Other common mental disorders: global health estimates. Geneva: World Health Organization, 24.

[ref18] Edurne Z. D. O. Pombo H. Pons Vigués M. Casajuana Closas M. Motrico Martínez E. Moreno Peral P. (2018). Complex multiple risk intervention topromote healthy behaviours in peoplebetween 45 to 75 years attended inprimary health care (EIRA study): study protocol for a hybrid trial. BMC Public Health 18, 1–15. doi: 10.1186/s12889-018-5805-y

[ref19] Fachado A. Menéndez M. González L. (2013). Apoyo social: mecanismos y modelos de influencia sobre la enfermedad crónica. Cad. Aten. Primaria 19, 118–123.

[ref20] Ferrari A. J. Charlson F. J. Norman R. E. Patten S. B. Freedman G. Murray C. J. L.. (2013). Burden of depressive disorders by country, sex, age, and year: findings from the global burden of disease study 2010. PLoS Med. 10:e1001547. doi: 10.1371/journal.pmed.1001547, PMID: 24223526 PMC3818162

[ref21] Fontán B. Pérula de Torres L. Á. Navarro C. (2013). Current evidence on the motivational interview in the approach to health care problems in primary care. Aten. Primaria 45, 486–495. doi: 10.1016/j.aprim.2013.01.014, PMID: JM BF, Brun B, JA PC 24042074 PMC6985504

[ref22] Fuhrer R. Stansfeld S. A. Chemali J. Shipley M. J. (1999). Gender, social relations and mental health: prospective findings from an occupational cohort (Whitehall II study). Soc. Sci. Med. 48, 77–87. doi: 10.1016/s0277-9536(98)00290-1, PMID: 10048839

[ref23] Fusilier M. R. Ganster D. C. Mayes B. T. (1986). The social support and health relationship: is there a gender difference? J. Occup. Psychol. 59, 145–153. doi: 10.1111/j.2044-8325.1986.tb00220.x

[ref24] Gabilondo Cuéllar A. Rojas Farreras S. Vilagut Saiz G. Haro Abad J. M. Fernández Sánchez A. Pinto Meza A. A.. (2010). Epidemiology of major depressive episode in a southern European country: results from the ESEMeD-Spain project. J. Affect. Disord. 120, 76–85. doi: 10.1016/j.jad.2009.04.016, PMID: 19428121 PMC3756284

[ref25] Ganster D. C. Victor B. (1988). The impact of social support on mental and physical health. Br. J. Med. Psychol. 61, 17–36. doi: 10.1111/j.2044-8341.1988.tb02763.x, PMID: 3282536

[ref26] Gariépy G. Honkaniemi H. Quesnel-Vallee A. (2016). Social support and protection from depression: systematic review of current findings in Western countries. Br. J. Psychiatry 209, 284–293. doi: 10.1192/bjp.bp.115.169094, PMID: 27445355

[ref27] Geerlings S. W. Beekman A. T. F. Deeg D. J. H. Van Tilburg W. (2000). Physical health and the onset and persistence of depression in older adults: an eight-wave prospective community-based study. Psychol. Med. 30, 369–380. doi: 10.1017/s0033291799001890, PMID: 10824657

[ref28] Golden J. Conroy R. M. Bruce I. Denihan A. Greene E. Kirby M.. (2009). Loneliness, social support networks, mood and wellbeing in community-dwelling elderly. Int. J. Geriatric Psychiatry 24, 694–700. doi: 10.1002/gps.2181, PMID: 19274642

[ref29] Gómez-Gómez I. Motrico E. Moreno-Peral P. Casajuana-Closas M. López-Jiménez T. Zabaleta-del-Olmo E.. (2023). A multiple health behaviour change intervention to prevent depression: a randomized controlled trial. Gen. Hosp. Psychiatry 82, 86–94. doi: 10.1016/j.genhosppsych.2023.02.004, PMID: 37001428

[ref30] Häusser J. Abdel Hadi S. Reichelt C. Mojzisch A. J. (2023). The reciprocal relationship between social identification and social support over time: a four-wave longitudinal study. Br. J. Soc. Psychol. 62, 456–466. doi: 10.1111/bjso.12553, PMID: 35758709

[ref31] Hayes A. F. Preacher K. J. (2014). Statistical mediation analysis with a multicategorical independent variable. Br. J. Math. Stat. Psychol. 67, 451–470. doi: 10.1111/bmsp.12028, PMID: 24188158

[ref32] Herdman M. Badia X. Berra S. (2001). El EuroQol-5D: una alternativa sencilla para la medición de la calidad de vida relacionada con la salud en atención primaria. Atención Primaria 28, 425–429. doi: 10.1016/S0212-6567(01)70406-4, PMID: 11602124 PMC7684037

[ref33] Hernan M. A. Robins J. (2020). Causal inference: what if. Boca Raton: Chapman & Hill/CRC.

[ref34] Holt-Lunstad J. Smith T. B. Layton J. B. (2010). Social relationships and mortality risk: a meta-analytic review. PLoS Med. 7:e1000316. doi: 10.1371/journal.pmed.1000316, PMID: 20668659 PMC2910600

[ref35] Hoogeveen R. C. Dorresteijn J. A. N. Kriegsman D. M. W. Valk G. D. (2015). Complex interventions for preventing diabetic foot ulceration. Cochrane Database Syst. Rev. 2015:CD007610. doi: 10.1002/14651858.cd007610.pub3, PMID: 26299991 PMC8504983

[ref36] Hosseini Z. Safari A. Khan N. A. Veenstra G. Conklin A. I. (2021). Gender differences in the role of social support for hypertension prevention in Canada: a population-based cross-sectional study of the Canadian longitudinal study on aging cohort. CJC open 3, S62–S70. doi: 10.1016/j.cjco.2021.09.016, PMID: 34993435 PMC8712674

[ref37] Hyde J. S. Mezulis A. H. Abramson L. Y. (2008). The ABCs of depression: integrating affective, biological, and cognitive models to explain the emergence of the gender difference in depression. Psychol. Rev. 115, 291–313. doi: 10.1037/0033-295x.115.2.291, PMID: 18426291

[ref38] Jaehn P. Bobrova N. Saburova L. Kudryavtsev A. V. Malyutina S. Cook S. (2020). The relation of gender role attitudes with depression and generalised anxiety disorder in two Russian cities. J. Affect. Disord. 264, 348–357. doi: 10.1016/j.jad.2020.01.027, PMID: 32056771

[ref39] Jylhä M. (2009). What is self-rated health and why does it predict mortality? Towards a unified conceptual model. Soc. Sci. Med. 69, 307–316. doi: 10.1016/j.socscimed.2009.05.013, PMID: 19520474

[ref40] Kim S. Jang Y. S. Park E.-C. (2016). Direct and indirect effects of five factor personality and gender on depressive symptoms mediated by perceived stress. PLoS One 11:e0154140. doi: 10.1371/journal.pone.0154140, PMID: 27120051 PMC4847785

[ref41] Kim S. Jang Y. S. Park E.-C. (2025). Associations between social isolation, withdrawal, and depressive symptoms in young adults: a cross-sectional study. BMC Psychiatry 25, 327–312. doi: 10.1186/s12888-025-06792-6, PMID: 40181348 PMC11966788

[ref42] Koster A. Bosma H. Kempen G. I. J. M. Penninx B. W. J. H. Beekman A. T. F. Deeg D. J. H.. (2006). Socioeconomic differences in incident depression in older adults: the role of psychosocial factors, physical health status, and behavioral factors. J. Psychosom. Res. 61, 619–627. doi: 10.1016/j.jpsychores.2006.05.009, PMID: 17084139

[ref43] Kumparatana P. Cournos F. Terlikbayeva A. Rozental Y. Gilbert L. P. (2017). Factors associated with self-rated health among migrant workers: results from a population-based cross-sectional study in Almaty, Kazakhstan. Int. J. Public Health 62, 541–550. doi: 10.1007/s00038-017-0944-y, PMID: 28233019 PMC5429909

[ref44] Leskelä U. S. Melartin T. K. Lestelä-Mielonen P. S. Rytsälä H. J. Sokero T. P. Heikkinen M. E.. (2004). Life events, social support, and onset of major depressive episode in Finnish patients. J. Nerv. Ment. Dis. 192, 373–381. doi: 10.1097/01.nmd.0000126705.15497.c9, PMID: 15126892

[ref45] Lima-Costa M. F. Firmo J. O. A. Uchôa E. (2005). Diferenças na estrutura da auto-avaliação da saúde em idosos com diferente situação sócio-econômica: Projeto Bambuí. Cad. Saude Publica 21, 830–839. doi: 10.1590/S0102-311X2005000300017, PMID: 15868041

[ref46] Lin J. Zou L. Lin W. Becker B. Yeung A. Cuijpers P.. (2021). Does gender role explain a high risk of depression? A meta-analytic review of 40 years of evidence. J. Affect. Disord. 294, 261–278. doi: 10.1016/j.jad.2021.07.018, PMID: 34304081

[ref47] Lino V. T. S. Portela M. C. Camacho L. A. B. Atie S. Lima M. J. Z. (2013). Assessment of social support and its association to depression, self-perceived health and chronic diseases in elderly individuals residing in an area of poverty and social vulnerability in Rio de Janeiro City, Brazil. PLoS One 8:e71712. doi: 10.1371/journal.pone.0071712, PMID: 23951227 PMC3741124

[ref48] Lorant V. Deliège D. Eaton W. Robert A. Philippot P. Ansseau M. (2003). Socioeconomic inequalities in depression: a meta-analysis. Am. J. Epidemiol. 157, 98–112. doi: 10.1093/aje/kwf182, PMID: 12522017

[ref49] Manea L. Gilbody S. McMillan D. (2015). A diagnostic meta-analysis of the patient health Questionnaire-9 (PHQ-9) algorithm scoring method as a screen for depression. Gen. Hosp. Psychiatry 37, 67–75. doi: 10.1016/j.genhosppsych.2014.09.009, PMID: 25439733

[ref50] Mc Sharry J. Olander E. K. French D. P. (2015). Do single and multiple behavior change interventions contain different behavior change techniques? A comparison of interventions targeting physical activity in obese populations. Health Psychol. 34, 960–965. doi: 10.1037/hea0000185, PMID: 25528182

[ref51] Moustafa A. A. Crouse J. J. Herzallah M. M. Salama M. Mohamed W. Misiak B.. (2020). Depression following major life transitions in women: a review and theory. Psychol. Rep. 123, 1501–1517. doi: 10.1177/0033294119872209, PMID: 31470771

[ref52] Nicolosi G. T. Falcão D. V. Batistoni S. S. Lopes A. Cachioni M. Neri A. L.. (2011). Depressive symptoms in old age: relations among sociodemographic and self-reported health variables. Int. Psychogeriatr. 23, 941–949. doi: 10.1017/S1041610211000627, PMID: 21486519

[ref53] Payne J. Coy J. Milner P. Patterson S. (1993). Are deprivation indicators a proxy for morbidity? A comparison of the prevalence of arthritis, depression, dyspepsia, obesity and respiritory symptoms with unemployment rates and Jarman scores. J. Public Health Med. 1, 161–171. doi: 10.1007/bf02959657, PMID: 8353006

[ref54] Petkovic J. Duench S. Trawin J. Dewidar O. Pardo J. P. Simeon R. J.. (2021). Behavioural interventions delivered through interactive social media for health behaviour change, health outcomes, and health equity in the adult population. Cochrane Database Syst. Rev. 2021:CD012932. doi: 10.1002/14651858.cd012932.pub2, PMID: 34057201 PMC8406980

[ref55] Plaisier I. de Bruijn J. G. M. de Graaf R. ten Have M. Beekman A. T. F. Penninx B. W. J. H. (2007). The contribution of working conditions and social support to the onset of depressive and anxiety disorders among male and female employees. Soc. Sci. Med. 64, 401–410. doi: 10.1016/j.socscimed.2006.09.008, PMID: 17055138

[ref56] Red de Investigación en Actividades Preventivas y Promoción de la Salud (n.d.). Available online at: www.rediapp.org

[ref57] Sa S. (2003). Social inequalities in depressive symptoms and physical functioning in the Whitehall II study: exploring a common cause explanation. J. Epidemiol. Community Health 57, 361–367. doi: 10.1136/jech.57.5.361, PMID: 12700221 PMC1732450

[ref58] Salk R. H. Hyde J. S. Abramson L. Y. (2017). Gender differences in depression in representative national samples: Meta-analyses of diagnoses and symptoms. Psychol. Bull. 143, 783–822. doi: 10.1037/bul0000102, PMID: 28447828 PMC5532074

[ref59] Scott K. M. Bruffaerts R. Tsang A. Ormel J. Alonso J. Angermeyer M. C.. (2007). Depression–anxiety relationships with chronic physical conditions: results from the world mental health surveys. J. Affect. Disord. 103, 113–120. doi: 10.1016/j.jad.2007.01.015, PMID: 17292480

[ref60] Sobocki P. Jönsson B. Angst J. Rehnberg C. P. (2006). Cost of depression in Europe. J. Ment. Health Policy Econ. 9, 87–98, PMID: 17007486

[ref61] Stanton A. M. Chiu C. Dolotina B. Kirakosian N. King D. S. Grasso C.. (2025). Disparities in depression and anxiety at the intersection of race and gender identity in a large community health sample. Soc. Sci. Med. 365:117582. doi: 10.1016/j.socscimed.2024.117582, PMID: 39631299 PMC13052829

[ref62] Sun Q. Shen X. Qi M. Suliman M. Tian S. (2024). The mediating role of interoceptive sensitivity in the relationship between physical activity and depression symptoms in college students. Behav. Sci. 14:608. doi: 10.3390/bs14070608, PMID: 39062431 PMC11273503

[ref63] Surkan P. J. O’Donnell E. M. Berkman L. F. Peterson K. E. (2009). Social ties in relation to health status of low-income Brazilian women. J. Women's Health 18, 2049–2056. doi: 10.1089/jwh.2008.1340, PMID: 20044869 PMC2864470

[ref65] Thoits P. A. (1982). Conceptual, methodological, and theoretical problems in studying social support as a buffer against life stress. J. Health Soc. Behav. 23, 145–159. doi: 10.2307/2136511, PMID: 7108180

[ref66] Thoits P. A. (1992). Identity structures and psychological well-being: gender and marital status comparisons. Soc. Psychol. Q. 55, 236–256. doi: 10.2307/2786794

[ref67] Thompson R. A. Flood M. F. Goodvin R. (2015). “Social support and developmental psychopathology” in Developmental psychopathology: Volume three: Risk, disorder, and adaptation. John Wiley & Sons, Inc. 1–37.

[ref68] Wang J. Mann F. Lloyd-Evans B. Ma R. Johnson S. (2018). Associations between loneliness and perceived social support and outcomes of mental health problems: a systematic review. BMC Psychiatry 18, 156–116. doi: 10.1186/s12888-018-1736-5, PMID: 29843662 PMC5975705

